# Social competition as a driver of phenotype–environment correlations: implications for ecology and evolution

**DOI:** 10.1111/brv.12768

**Published:** 2021-06-18

**Authors:** Rienk W. Fokkema, Peter Korsten, Tim Schmoll, Alastair J. Wilson

**Affiliations:** ^1^ Department of Animal Behaviour Bielefeld University Konsequenz 45 Bielefeld 33615 Germany; ^2^ Evolutionary Biology Bielefeld University Konsequenz 45 Bielefeld 33615 Germany; ^3^ Conservation Ecology Group, Groningen Institute for Evolutionary Life Sciences (GELIFES) University of Groningen Nijenborgh 7 Groningen 9747AG The Netherlands; ^4^ Centre for Ecology and Conservation University of Exeter (Penryn Campus) Penryn Cornwall TR10 9FE United Kingdom

**Keywords:** intraspecific competition, phenotype–environment correlation, fitness variation, individual quality, habitat quality, adaptation, microevolution, indirect genetic effect (IGE), evolutionary stasis

## Abstract

While it is universally recognised that environmental factors can cause phenotypic trait variation *via* phenotypic plasticity, the extent to which causal processes operate in the reverse direction has received less consideration. In fact individuals are often active agents in determining the environments, and hence the selective regimes, they experience. There are several important mechanisms by which this can occur, including habitat selection and niche construction, that are expected to result in phenotype–environment correlations (i.e. non‐random assortment of phenotypes across heterogeneous environments). Here we highlight an additional mechanism – intraspecific competition for preferred environments – that may be widespread, and has implications for phenotypic evolution that are currently underappreciated. Under this mechanism, variation among individuals in traits determining their competitive ability leads to phenotype–environment correlation; more competitive phenotypes are able to acquire better patches. Based on a concise review of the empirical evidence we argue that competition‐induced phenotype–environment correlations are likely to be common in natural populations before highlighting the major implications of this for studies of natural selection and microevolution. We focus particularly on two central issues. First, competition‐induced phenotype–environment correlation leads to the expectation that positive feedback loops will amplify phenotypic and fitness variation among competing individuals. As a result of being able to acquire a better environment, winners gain more resources and even better phenotypes – at the expense of losers. The distinction between individual quality and environmental quality that is commonly made by researchers in evolutionary ecology thus becomes untenable. Second, if differences among individuals in competitive ability are underpinned by heritable traits, competition results in both genotype–environment correlations and an expectation of indirect genetic effects (IGEs) on resource‐dependent life‐history traits. Theory tells us that these IGEs will act as (partial) constraints, reducing the amount of genetic variance available to facilitate evolutionary adaptation. Failure to recognise this will lead to systematic overestimation of the adaptive potential of populations. To understand the importance of these issues for ecological and evolutionary processes in natural populations we therefore need to identify and quantify competition‐induced phenotype–environment correlations in our study systems. We conclude that both fundamental and applied research will benefit from an improved understanding of when and how social competition causes non‐random distribution of phenotypes, and genotypes, across heterogeneous environments.

## I. INTRODUCTION

Phenotypic variation within natural populations is often non‐randomly distributed with respect to the heterogeneity of the environment. Such phenotype–environment correlation is expected given the ubiquity of phenotypic plasticity: the causal effect of environmental factors on trait expression of a genotype (Pigliucci, 2001, 2005; Nussey, Wilson & Brommer, 2007). Thus even genetically identical individuals will tend to diverge from an initially similar phenotype if exposed to spatial or temporal differences in the environment (Pigliucci, 2005; Dingemanse *et al*., 2010; Sultan, 2015; Table 1). However, less recognised is that causality can also operate in the opposite direction (Saltz & Nuzhdin, 2014; Edelaar & Bolnick, 2019). This occurs when individuals are active agents in determining the environmental conditions they experience (Edelaar & Bolnick, 2012, 2019; Saltz & Nuzhdin, 2014; Laland *et al*., 2015; Sultan, 2015; Müller *et al*., 2020). For instance, individuals may differ (genetically or non‐genetically) in habitat preference, and/or may modify the conditions of their immediate environment to suit their specific needs (see schematic overview in Edelaar & Bolnick, 2019; Table 1).

**Table 1:** Overview of the key causal processes by which phenotype (P)–environment (E) correlations can occur within natural populations. The direction of causality may initially be E➔P (plasticity, viability selection) or P➔E (habitat choice, habitat construction, social competition). However, in the latter case subsequent feedback loops are likely for plastic phenotypic traits (E➔P). In this review we focus specifically on the process of social competition, highlighting its consequences for the evolutionary dynamics of natural populations. The environment/niche includes both abiotic and biotic aspects. We refer the interested reader to Edelaar & Bolnick (2019) for an alternative but similar scheme and a more in depth treatment of other mechanisms than social competition

Process	Description	Causality	Can result in genotype–environment correlation?	Example references
Phenotypic plasticity	The causal effect of environmental factors on phenotypic trait expression. Through this process the same genotype can express a range of phenotypes depending on the environment	E 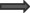 P	No	West‐Eberhard (1989); Pigliucci (2005)
Divergent selection	Individuals within the population initially distribute randomly across environments, but environment‐specific patterns of selective mortality then generate phenotype–environment correlation	E 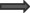 P (through viability selection)	Yes	Hendry (2004); Reznick *et al*. (2008)
Habitat/niche choice	The non‐random distribution of individual phenotypes over the environment based on (genetic) differences in their habitat preferences	P 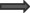 E, but after that E 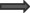 P (through plasticity)	Yes	Edelaar & Bolnick (2012, 2019)
Habitat/niche construction	Individual phenotypes may (genetically) differ in how they modify their immediate environment to suit their specific needs	P 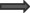 E, but after that E 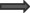 P (through plasticity)	Yes	Saltz & Nuzhdin (2014); Sultan (2015); Edelaar & Bolnick (2019)
Social competition	Individuals may share a habitat preference, but social competition can result in some individuals being excluded, resulting in phenotype–environment correlations based on (genetic) differences in competitive ability	P 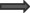 E, but after that E 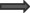 P (through plasticity)	Yes	Wilson (2014); Fisher & McAdam (2019)

Variation in habitat preference and resulting habitat choice is perhaps the most obvious mechanism which leads to a non‐random distribution of phenotypes across heterogeneous environments. An illustrative example of matching habitat choice (e.g. Edelaar, Siepielski & Clobert, 2008; Camacho *et al*., 2020; for a useful discussion of the different mechanisms that can underlie habitat choice see Akcali & Porter, 2017), is a study on dunnocks (*Prunella modularis*) where bold individuals (measured by a low flight‐initiation distance in response to human observers) were shown to reside more in areas with higher levels of human disturbance, whereas shy individuals preferred areas with lower levels of disturbance (Holtmann *et al*., 2017). Although the direction of causality was not proved here, the authors argue that intrinsic differences in boldness determine the distribution of dunnocks across environments rather than *vice versa*, and that behavioural plasticity is unlikely to account for the observed patterns (Holtmann *et al*., 2017; for a similar case example see Pearish, Hostert & Bell, 2013). Ultimately, differences in habitat preference could even lead to population subdivision, restricting gene flow and facilitating evolutionary divergence. For instance, in three‐spined sticklebacks (*Gasterosteus aculeatus*), Bolnick *et al*. (2009) demonstrated that populations in adjacent stream and lake habitats were both morphologically and genetically divergent. Here, a translocation experiment revealed a strong phenotype‐dependent habitat preference, suggesting that behavioural preference may well be important in maintaining (and perhaps initiating) population differentiation. The latter could occur through divergent selection (Hendry, Taylor & McPhail, 2002; Hendry, 2004; Reznick, Ghalambor & Crooks, 2008; Räsänen *et al*., 2012), which itself could also act as an independent driver of phenotype–environment correlations within populations (see Table 1).

However, variation in habitat preference is not a necessary condition for causal phenotype‐to‐environment relationships to arise. If high‐quality habitat patches are preferred by all phenotypes, but their availability is limited, social competition (here defined as competition among the set of conspecifics interacting in a social environment) can result in some individuals being excluded from them (Table 1). Thus, particularly in territorial species, individuals that are stronger competitors will monopolize high‐quality habitat patches at the expense of others (Bernstein, Krebs & Kacelnik, 1991; Newton, 1998; Ward, Webster & Hart, 2006; van de Pol *et al*., 2007; for a notable theoretical treatment see Baldauf, Engqvist & Weissing, 2014). Consequently, among‐individual differences in any traits conferring competitive ability will lead to phenotype–environment correlations. Patch quality obtained may then have secondary effects *via* phenotypic plasticity. If traits conferring a competitive advantage are also phenotypically plastic, then this could generate positive feedback loops that amplify phenotypic and fitness variation among individuals. As a simple illustration, if body size determines competitive outcomes, initial size differences will mean larger individuals claim better patches with more food resources, which will in turn allow faster growth and so generate greater asymmetry in future competitiveness.

We believe this scenario, in which phenotype–environment correlations are caused by among‐individual differences in competitive ability, is likely to be common and has major, but poorly appreciated, implications for ecology and evolution. For current purposes we view competition as distinct, although often related, to other mechanisms generating phenotype–environment correlations in natural populations [for their in‐depth treatment see, for example, Edelaar & Bolnick (2012, 2019), Baldauf *et al*. (2014), Saltz & Nuzhdin (2014) and Nicolaus & Edelaar (2018)]. To enable this distinction, and so our focus on competition, we implicitly consider the ‘quality’ of a habitat patch to be defined by characteristics over and above the level of competition. Thus, we can envisage a simple scenario for illustration purposes in which all individuals share a common preference (e.g. for high‐quality habitat) but only strong competitors can realise it. We refer the reader to Edelaar & Bolnick (2019) for an alternative perspective that emphasises habitat selection rather than competition as a driver of phenotype–environment correlation. In their view it may be argued that individuals with less‐competitive phenotypes select the habitat patches that are best for them given the social context (e.g. by preferring patches with fewer competitors).

Herein we argue that empirical studies have often failed to consider the implications of competition‐induced phenotype–environment correlations appropriately, and raise four key questions that we believe need to be addressed both conceptually and empirically. For each question we summarise and evaluate the empirical results gathered to date and discuss promising methodological approaches that may help us to reach more definitive answers. First, we ask how prevalent competition‐induced phenotype–environment correlations actually are. Second, we ask what they mean for the common distinction made by evolutionary ecologists between individual and environmental ‘quality’. Third, we ask whether positive feedback loops (as outlined above) are likely to be an important source of fitness variation in wild populations. Finally, we ask what consequences this phenomenon might have for the evolutionary dynamics of phenotypes under selection. We conclude that in fact competition for high‐quality habitat may be a hidden source of evolutionary constraint that goes some way towards explaining the frequent observation of evolutionary stasis in heritable traits under directional selection (Merilä, Sheldon & Kruuk, 2001; Pujol *et al*., 2018).

## II. DOES SOCIAL COMPETITION DRIVE PHENOTYPE–ENVIRONMENT CORRELATIONS IN NATURAL POPULATIONS?

**Fig 1:**
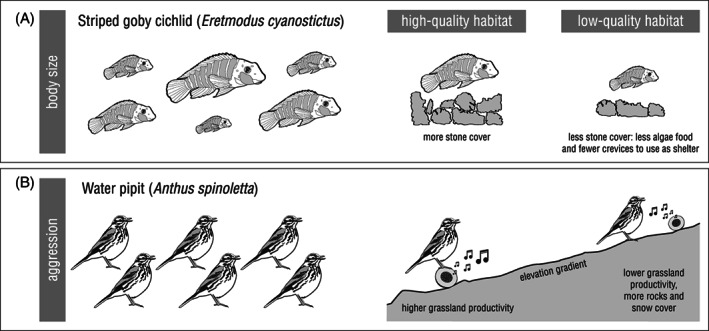
Two examples of studies on natural populations where social competition‐induced phenotype–environment correlations were detected. Positive correlations were found between indices of habitat quality and body size (A; Taborsky *et al*., 2014) and aggression (B; approach distance to conspecific territorial song playback; Bastianelli *et al*., 2015), respectively.

Individuals with phenotypic characteristics likely to give them a competitive advantage are often found in higher quality habitat patches (Fig. 1). Such characteristics can for example include large body size (e.g. Verhulst, Perrins & Riddington, 1997; Sergio *et al*., 2009; Taborsky, Guyer & Demus, 2014) or high aggressiveness (e.g. Snekser *et al*., 2009; Morales *et al*., 2014; Bastianelli *et al*., 2015). While this pattern is consistent with social competition driving the environmental conditions experienced by individuals, it is not conclusive evidence. Positive correlations between habitat quality and (putative) phenotypic indicators of competitive ability are not always detected [e.g. size (Ens, Weissing & Drent, 1995); aggression (Stehle *et al*., 2017)]. Furthermore, even where predicted phenotype–environment correlations are detected, it may not be possible to exclude the possibility that they arise from phenotypic plasticity (following dispersal, movement, and settlement processes that are all random with respect to the initial phenotype). For instance, arriving at unoccupied patches may be random with respect to phenotype, but information gained about patch quality following arrival may influence how aggressively (and successfully) it is defended against rivals (e.g. Snekser *et al*., 2009).

Experiments are therefore vital to establish causation underpinning observed phenotype–environment correlations. A useful example is provided by Studds & Marra (2005) who studied wintering habitat use in American redstarts (*Setophaga ruticilla*) by removing behaviourally dominant adult males from high‐quality mangrove habitat patches. This resulted in immature males and females moving into the mangroves from shrub habitat, demonstrating that all demographic groups actually share the preference for mangroves but that some are normally excluded. Earlier work suggests that this pattern may be partly driven by differences in body mass among these demographic groups (Marra, 2000). Interestingly, the experimentally ‘upgraded’ birds that moved into better habitat started spring migration earlier and had a higher return rate the following winter relative to control birds (Studds & Marra, 2005). This shows how social competition for habitat patches can not only cause phenotype–environment correlations but also generate downstream carry‐over effects (through phenotypic plasticity) on fitness‐related traits expressed later.

Intuitively, we might expect competition‐induced phenotype–environment correlations to be most prevalent in territorial systems or where individuals engage in contest competition over discrete habitat patches (Bernstein *et al*., 1991; Grand & Grant, 1994; Newton, 1998; Ward *et al*., 2006). However, analogous situations can arise over more continuous spatial scales. For instance, in colonial breeding species, more competitive individuals can claim safer space in the core of the colony, relegating others to the margins where predation risk is higher (Minias, 2014).

Another important line of evidence that differential competitive ability leads to phenotype–environment correlations comes from empirical tests of the ‘Ideal Despotic Distribution’ (Fretwell, 1972; Parker & Sutherland, 1986). In this model, dominant individuals prevent others from settling nearby, ensuring local competitor density does not become high enough to suppress their own performance. This model effectively predicts observed distributions in natural populations of various species (e.g. Bernstein *et al*., 1991; Mosser *et al*., 2009; Falcy, 2015). Unsurprisingly, more competitive individuals in high‐quality patches with low competitor density also obtain higher fitness (e.g. Sergio *et al*., 2007; Mosser *et al*., 2009).

Overall, the above‐mentioned work provides strong indications that phenotype–environment correlations are common in natural populations. The present evidence is however far from conclusive and experimental studies in particular could importantly contribute to demonstrating the existence and measuring the strength of such correlations.

## III. CAN WE SEPARATE THE ‘QUALITY’ OF AN INDIVIDUAL FROM THAT OF ITS ENVIRONMENT?

**Fig 2:**
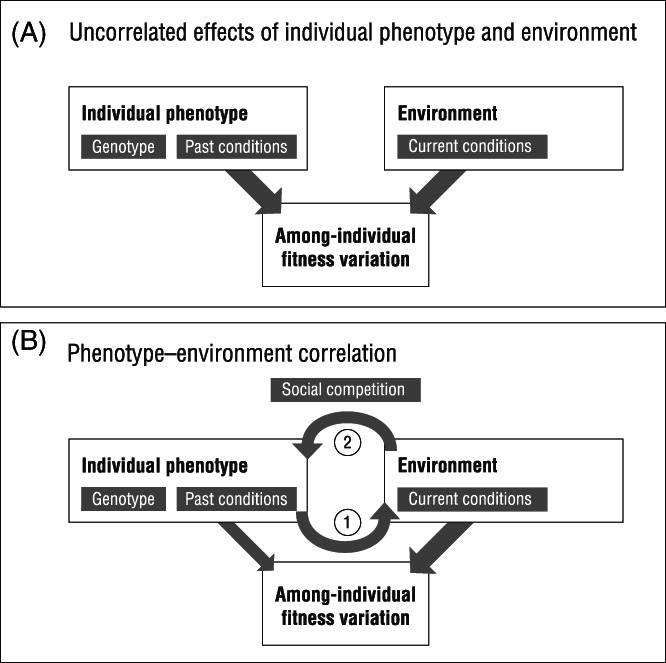
(A) The contributions of individual phenotype and environment to among‐individual fitness variation are often (implicitly) assumed to be uncorrelated. (B) However, in natural populations, through social competition, effects of the individual phenotype may act mostly indirectly *via* the probability of individuals obtaining high‐quality habitat patches (arrow 1: distribution across the environment). Moreover, small differences in the initial success of individuals in obtaining high‐quality habitat patches could in turn lead to positive feedback loops between individual phenotypes and the environment (arrow 2: phenotypic plasticity; leading to arrow 1 again). As a result of being in a better environment, winners gain better phenotypes – and so increased fitness – at the expense of losers, thus amplifying among‐individual fitness variation.

Concepts of individual and habitat quality are widely used in the fields of ecology and evolution and typically treated as separable (e.g. Pettorelli *et al*., 2001; Johnson, 2007; Mosser *et al*., 2009; Wilson & Nussey, 2010; Bergeron *et al*., 2011; Germain & Arcese, 2014). In general, researchers often (implicitly) assume that contributions to observed variation in phenotypic traits or fitness measures come from both ‘intrinsic’ and ‘extrinsic’ sources (e.g. Harrison *et al*., 2011; Daunt *et al*., 2014; Walsh *et al*., 2016). At least when applied to traits closely linked to fitness, the former is sometimes conceptualised as ‘among‐individual variation in quality’ (Wilson & Nussey, 2010). The latter might then capture extrinsic abiotic and biotic (including social) environmental factors that can collectively be conceptualised as ‘environmental quality’. This distinction is convenient and, by assuming that ‘intrinsic’ and ‘extrinsic’ effects are independent and work additively to determine trait expression, they are in principle separately estimable in empirical studies (Charmantier, Garant & Kruuk, 2014; Fig. 2A). This means we can, for instance, explore the causes and consequences of (intrinsic) among‐individual fitness variation while controlling for (extrinsic) variation in environmental quality.

So how do phenotype–environment correlations complicate this picture? Crucially, and regardless of how the correlations arise, they create a statistical problem in the sense that intrinsic and extrinsic effects become collinear (Fig. 2B), which means that they are harder to separate in real‐world data sets. To date, those empirical studies explicitly recognising phenotype–environment correlations have largely done so within the context of trying to obtain unbiased estimates of individual and environmental contributions to observed fitness variation (Studds & Marra, 2005; Sergio *et al*., 2009; Germain & Arcese, 2014; Mathot, Dekinga & Piersma, 2017; Fokkema, Ubels & Tinbergen, 2018). A recent example is provided by Pärt *et al*. (2017) who investigated why breeding late in the season is associated with reduced fitness (a common finding in iteroparous animal populations; see Verhulst & Nilsson, 2008). More specifically, they asked, using a study population of northern wheatears (*Oenanthe oenanthe*), whether (*i*) low fitness is causally dependent on later breeding *per se* (i.e. direct selection on breeding time), (*ii*) late breeders are of lower intrinsic individual quality (such that breeding time is under indirect selection but fitness depends causally on unknown traits comprising quality), or (*iii*) environmental quality, and so fitness, is lower for later breeders because the best resourced territories have already been claimed. Note that this third possibility also posits direct selection on breeding time, but additionally, and in contrast to mechanism (*i*), argues that the causal trait–fitness pathway operates through environmental quality as an intermediate. Using variance–covariance decomposition to partition the relationships among laying date of the first egg and fitness traits into components attributable to individual (breeding female) and territory identities, the authors found some support for all three scenarios, but argued pathway (*i*) (direct selection on breeding time) had the strongest influence on fitness.

Statistical variance decomposition such as used in the Pärt *et al*. (2017) study described above may provide many useful insights, although as the authors themselves point out, robustly distinguishing between causal effects of female (intrinsic) quality and a pattern of deteriorating environmental quality across the season would be greatly facilitated by experimental manipulation of breeding time [Pärt *et al*., 2017; see Verhulst & Nilsson (2008) for a review of such experiments]. Where experimental manipulations are not possible, approaches such as path analysis and structural equation modelling (Grace *et al*., 2012; Shipley, 2016; Henshaw, Morrissey & Jones, 2020) may help to test and discriminate between competing causal hypotheses for relationships among phenotype, environment and fitness. Clearly, where experimentation is possible, coupling manipulation to appropriate data analysis offers the most powerful approach to disentangling individual phenotype (intrinsic) from environment (extrinsic) effects on fitness. This assumes that phenotype, fitness and environment are distinct but correlated entities linked by causal processes. Phenotypes influence fitness while environments can influence fitness directly but also *via* effects on phenotype (plasticity). However, this view is complicated by the presence of phenotype–environment correlations caused by processes such as social competition (phenotype → environment). In such a scenario, the assumption that individual and environmental quality make distinct, additive contributions to fitness variation is no longer tenable (Fig. 2B).

Consequently, we may need to re‐think what evolutionary inferences can be drawn from studies that seek to separate individual from environmental quality effects in the (known or possible) presence of causal phenotype–environment correlations. If we are willing to assume that, in general, phenotype–environment correlations are driven by plasticity, then our existing frameworks for modelling individual‐by‐environment interactions (I × E; i.e. among‐individual differences in plasticity) could provide a solution (e.g. Nussey *et al*., 2007; van de Pol & Wright, 2009; Dingemanse & Dochtermann, 2013; Westneat, Wright & Dingemanse, 2015). These explicitly recognise that traits – and fitness – can depend on interactions of intrinsic and extrinsic factors, and that adaptation can occur through the evolution of plasticity (under the assumption that individual‐by‐environment interactions have a heritable basis, i.e. are underpinned by genotype‐by‐environment interactions). However, if trait‐mediated competitive interactions over patches drive phenotype–environment correlations as we suggest, then the evolutionary consequences, and the modelling solutions, are less clear.

The question posed in this section – can we separate the ‘quality’ of an individual from that of its environment? – is thus not trivial, but might broadly be answered as ‘no’ in the scenario that phenotype–environment correlations are present. As we discussed above, what to do about this then depends on the direction of causal pathways that give rise to this correlation. In the remaining sections, we focus on the consequences of phenotype to environment causality arising from competition. We argue that social competition for limited resources is a key determinant of among‐individual fitness variation and that this has important implications for the evolutionary dynamics of natural populations that are deserving of more empirical scrutiny.

## IV. WILL POSITIVE FEEDBACK LOOPS AMPLIFY FITNESS VARIATION AMONG COMPETING INDIVIDUALS?

A related complication that arises when seeking to make a conceptual – or empirical – distinction between individual (intrinsic) or environmental (extrinsic) quality variation, is that feedback processes are expected under social competition (Wilson, Grimmer & Rosenthal, 2013; Sih *et al*., 2015). Typically these will be positive, at least with respect to among‐individual phenotypic and fitness variation [but see for instance Ezenwa & Snider (2016) for an interesting scenario in which costs of holding territories in terms of parasite burdens actually generate negative feedback processes within individuals]. What we mean by this can be understood by returning to the hypothetical scenario suggested above. Imagine that small initial differences in body size cause differential ability to monopolize high‐resource patches, and that access to more resources allows faster growth. In this scenario, variation in competitive ability, and in body size, will increase over ecological time. Initial winners grow faster than initial losers and become ever more competitive (i.e. likely to win future contests) as they do so. As a result of being in a better environment, winners gain better phenotypes – and so increased fitness – at the expense of losers.

The presence of positive feedback loops like this means that small early‐life trait differences could be amplified into large fitness variation among individuals later on (Fig. 2B). This may be particularly true for organisms with size‐based dominance hierarchies coupled to indeterminate growth [e.g. many teleost fish species (Huntingford *et al*., 1990; Candolin & Voigt, 2001; Ward *et al*., 2006; Taborsky *et al*., 2014)]. Hakoyama & Iguchi (2001) provide an illustrative experimental demonstration of this. They induced competition for food among clones of the amago salmon (*Oncorhynchus masou ishikawae*) that did not differ detectably in initial size or dominance status. However, within‐group body mass variation then increased over time, as some individuals acquired more food and grew faster at the expense of others. The distribution of the fish across resource patches changed from a random to an ideal free distribution, and then to an ideal despotic distribution over a four‐week period (Hakoyama & Iguchi, 2001).

In a laboratory‐based experimental study such as that described above it is difficult to quantify fitness variation arising in an ecologically relevant way. Larger body size commonly leads to increased reproductive success in teleosts (Sloman & Armstrong, 2002; Paull *et al*., 2010), so it is probably reasonable to suggest that similar competition in wild fish would amplify among‐individual fitness variation. However, complementing laboratory experiments with experiments in free‐living populations is necessary to infer ecological relevance more robustly (Sloman & Armstrong, 2002). For instance, Mäntylä *et al*. (2015) forced passerine pied flycatcher (*Ficedula hypoleuca*) males to breed in randomly selected territories of varying quality by randomly opening up nestboxes in territories one by one to males arriving on the breeding grounds after their spring migration. Here the phenotype–environment correlation is experimentally reduced; not all high‐quality (i.e. early‐arriving) males are able to secure high‐quality territories, while some low‐quality males (i.e. later arrivals) are. While the purpose here was to test hypotheses about habitat preference, the approach could equally be applied to test the importance of social competition in driving fitness variation. Specifically, we would predict that after decoupling the positive relationship between competitive ability and territory quality, among‐individual variation in fitness should be reduced.

Thus, where differences in individual competitive ability lead to phenotype–environment correlations, we expect positive feedback to amplify fitness variation among individuals. What remains unknown is just how much of the observable fitness variation might actually be attributable to this phenomenon. Closing this gap in our knowledge is important for several reasons. First, understanding what maintains variation in fitness (and fitness‐related traits) in natural populations is arguably one of the central goals of evolutionary ecology. Second, the amount of variation in (relative) fitness defines the overall opportunity for natural selection on traits (Wade & Arnold, 1980). Third, since natural selection is a causal dependence of fitness on phenotype, estimating it using standard correlational approaches requires that no (important) traits are missed (Morrissey, Kruuk & Wilson, 2010). However, while estimates of natural selection on resource‐dependent traits (e.g. life‐history traits, growth) are abundant, empirical analyses have rarely included measures of competitive ability or social dominance that – under competition – are expected causally to drive differences in resource acquisition and so fitness (Wilson, 2014). This does not mean variable habitat quality in time or space has been completely ignored by selection studies; indeed the opposite is true precisely because environmentally induced phenotype–fitness covariance is widely recognised as a source of bias in selection estimates (Kruuk, Merilä & Sheldon, 2003). However, rather than being an ‘extrinsic’ nuisance variable to control for when estimating selection on phenotype, environmental quality may represent a useful proxy of the important (but unmeasured) trait of competitive ability (and could in fact be seen as part of the extended phenotype of the organism; discussed further in Section V).

## V. WHAT ARE THE CONSEQUENCES FOR EVOLUTIONARY DYNAMICS?

As described in Section IV, positive feedback under competition‐induced phenotype–environment correlations has implications for how we think about, and measure, selection on resource‐dependent traits affected by environmental quality. However, there are also implications for the amount of genetic variance present, and so the expected evolutionary responses to selection. This is particularly true when phenotype–environment correlations are also reflected on the genetic level by genotype–environment correlations (Saltz, 2011, 2019; Saltz & Nuzhdin, 2014). Such correlations exist if genotypes (not just phenotypes) are non‐randomly distributed within a heterogeneous environment. Note that the phenomenon of genotype–environment *correlation* is distinct from that of genotype‐by‐environment *interaction* (when genetic effects on the phenotype depend on the environment). In quantitative genetic equations both the presence of genotype‐by‐environment interactions (G × E) and genotype–environment correlations [cov(G, E)] can be accounted for [see box 2 in Saltz & Nuzhdin (2014) for a useful discssion of the latter term]. Traditional methods of estimating additive variance *V*_A_ (e.g. ANOVA) require an assumption that cov(G, E) is zero, and laboratory‐based quantitative genetic studies generally aim carefully to break down any genotype–environment correlation experimentally (e.g. by splitting families of fish across tanks; e.g. White & Wilson, 2019). Mixed‐model approaches to estimating *V*_A_ are more flexible, requiring an assumption that cov(G, E) is zero conditional on all other terms in the model. This means that statistical control is possible for environmental sharing by relatives, which represents one possible source of genotype–environment correlation and bias in *V*_A_.

Like phenotype–environment correlations, genotype–environment correlations can arise without a causal genotype‐to‐environment pathway. Specifically, limited dispersal can result in relatives being clustered within heterogeneous environments. When genetically similar individuals (i.e. relatives) tend to experience the same environments, phenotypic similarity truly caused by plasticity can be mistakenly attributed to shared genes (Kruuk & Hadfield, 2007). This is widely recognised as a possible source of upward bias when trying to estimate (additive) genetic variances for phenotypic traits (Kruuk *et al*., 2003; Stopher *et al*., 2012; Regan *et al*., 2017; Thomson *et al*., 2018; Evans, Postma & Sheldon, 2020). The solutions are to break the genotype–environment correlation experimentally (e.g. by cross‐fostering), or to incorporate shared environment effects into the statistical models used for estimating quantitative genetic parameters (e.g. Kruuk & Hadfield, 2007; Stopher *et al*., 2012; Regan *et al*., 2017; Thomson *et al*., 2018; Evans *et al*., 2020). Admittedly the first is not possible in all cases, while the second relies – as do all variance‐partitioning analyses – on appropriate data structure and volume. A case study of how modelling shared environment can impact estimates of genetic variance is provided by Stopher *et al*. (2012). Here, in a study of red deer (*Cervus elaphus*) on the Isle of Rum (Scotland), it was found that incorporating shared spatial effects reduced heritability (*h* ^2^) estimates of different ecologically relevant traits including, for example, rut home range size (for which estimated *h* ^2^ dropped from 31% to just 3%). However, such dramatic effects are neither observed nor expected in all cases [see Regan *et al*. (2017) for a counter‐example and discussion].

However, genotype–environment correlations can also reflect reversed causal pathways in which environments experienced by individuals depend on the expression of their genes. This has been long recognised in human psychiatry where twin and adoption studies have been used to test for genotype–environment correlations [see e.g. Jaffee & Price (2015) and Saltz (2019) for a review]. For example, children with genetic risk factors for antisocial behaviours were shown to experience a family environment with more harsh discipline imposed by their unrelated adoptive parents compared to adoptees not at genetic risk [for this and other examples see O'Connor *et al*. (1998) and Saltz (2019)]. Genotype–environment correlations viewed from this angle have received rather less attention to date in evolutionary ecology (but see e.g. Saltz & Nuzhdin, 2014). In general, genotype–environment relationships are likely whenever the environments experienced depend on the expression of heritable traits. These could be traits linked to habitat preference (Edelaar & Bolnick, 2012, 2019; Saltz & Nuzhdin, 2014; Nicolaus & Edelaar, 2018; Saltz, 2019), niche construction (Saltz & Nuzhdin, 2014; Edelaar & Bolnick 2019), or competitive ability (this review; Table 1). In these scenarios the ‘environment’ can be viewed as a heritable component of an ‘extended phenotype’ (Dawkins, 1982; Sultan, 2015; Hunter, 2018) and application of empirical approaches outlined above may actually cause downward bias in *V*_A_ for environmentally sensitive traits (rather than preventing upward bias as intended). Interestingly, as we explain in detail below, if heritable competitive ability differences drive the genotype–environment correlation it does not follow automatically that underestimating *V*_A_ means we also underestimate the potential for adaptive selection responses.

If among‐individual differences in the ability to obtain preferred habitat patches under competition have a genetic basis, genotype–environment correlations for fitness traits are expected. In simple terms, genes causing greater competitive ability will tend to be in good habitat patches. Heritable differences in competitive ability thus cause genetic variation in realised patch quality and, as a consequence, in resource‐dependent life‐history traits (Wilson, 2014). Importantly, however, not all the genetic variance in life‐history traits that is available will facilitate a response to selection. This is because of so‐called ‘indirect genetic effects’ (IGEs) that arise when the phenotype and fitness of an individual depend not just on its own genes, but also on genes carried by conspecifics, specifically competitors in our context (Bijma, 2011; Wilson, 2014; Costa e Silva *et al*., 2017; Schneider, Atallah & Levine, 2017; Bailey, Marie‐Orleach & Moore, 2018). For instance, imagine a population in which genetic factors carried by some individuals confer a higher than average competitive ability, and winning better quality territories allows earlier breeding which increases fitness. Here the ‘direct’ effect of genes carried by the good competitors will, all else being equal, predispose their bearers towards early breeding in good territories. However, the outcome of competitive interactions will also depend on ‘indirect’ effects of competitor genotypes. In any generation we expect more competitive genotypes to gain higher fitness by breeding early, but – despite breeding time being both heritable and under directional selection – mean breeding time may not evolve. The reason is that in each successive generation, the offspring of previous ‘winners’ must now compete against other winning lineages for the same availability of high‐quality territories (this phenomenon is also referred to as ‘evolutionary environmental deterioration’; Hadfield, Wilson & Kruuk, 2011). Put differently, despite both directional selection on and genetic variation for competitive ability and so breeding time, mean breeding time will not evolve if the availability of high‐quality environments is limited. We direct the interested reader to Wilson (2014) for a more mathematical review of the underlying quantitative genetic theory.

In the hypothetical population described above, evolution of traits that confer competitive ability (e.g. aggression) would proceed as expected, but the evolution of the trait that depends on competitive outcomes (i.e. breeding time) would be constrained (Fisher & McAdam, 2019). Consequently the population will always seem maladapted with respect to breeding time. Genetically determined differences in competitive ability thus offer a potential explanation for evolutionary stasis of heritable traits under directional selection, which is commonly observed in natural populations (Merilä *et al*., 2001; Pujol *et al*., 2018). In fact, while the contribution of competition‐driven IGE and genotype–environment correlation to evolutionary stasis remains to be studied, timing of reproduction does offer a very plausible case where this may be occurring. A warming climate means many seasonal breeders are under natural selection to advance their timing of reproduction to maintain synchrony with phenology of prey sources. Accordingly, advances in phenology are seen across many taxa (Parmesan, 2007; Thackeray *et al*., 2016; Cohen, Lajeunesse & Rohr, 2018). However, a recent meta‐analysis found consistent selection for earlier breeding across bird populations regardless of observed shifts in the timing of breeding over time (Radchuk *et al*., 2019). The interpretation that the authors pose is that focal populations are not getting better adapted because they are constantly lagging behind a shifting optimum. Alternatively, as Price, Kirkpatrick & Arnold (1988) suggested, selection could also be mostly acting on the non‐heritable environmental component of breeding time (i.e. the nutritional state of females; see also Kruuk *et al*., 2003). We add a third, but non‐exclusive possible interpretation here, and suggest that the apparent lag between selection and timing of breeding may result (at least in part) from evolutionary constraints imposed by competition over limiting high‐quality breeding habitat where earlier breeding can be achieved.

Determining whether social competition leads to causal genotype–environment correlations is thus important. If it does, the (direct) heritability of resource‐dependent life‐history traits (e.g. growth, fecundity, breeding time, survival) is generally expected to overestimate the potential for adaptive evolution. This expectation arises because of the ‘evolutionary environmental deterioration’ process that we outlined above. In IGE‐based quantitative genetic models, this process is reflected by a reduction in the total genetic variance available to selection (*sensu* Bijma, 2011) relative to the classically defined additive genetic variance (*V*_A_). The total genetic variance includes contributions from (direct) genetic effects, IGE and the covariance between them which – crucially for competitive interactions – will be negative. This is because genotypes that predispose to winning in competition when carried by a focal individual, predispose that focal individual to losing when encountered in a rival (Wilson, 2014).

There are two empirical strategies that offer a way forward where pedigree or genetic relatedness data makes quantitative genetic analysis possible. The first is to identify suitable proxies of competitive ability and test whether they are heritable, and if they are, to estimate genetic (co)variances for resource‐dependent life‐history traits conditional on competitive ability (Wilson, 2014). The logic behind this is that only the genetic variance that is independent of competitive outcomes will be free from the constraining indirect genetic effects, and thus available to facilitate adaptation. An alternative is to employ IGE models developed for livestock and forestry genetics (Ellen *et al*., 2014; Costa e Silva *et al*., 2017), and use these to characterise direct and indirect genetic parameters simultaneously, allowing estimation of the total genetic variance available to selection (sensu Bijma *et al*., 2007*a*; Bijma, Muir & Van Arendonk, 2007*b*).

A noteworthy recent example of empirical methods by which both the occurrence of indirect genetic effects under competition and their consequences for the rate of microevolution can be tested in natural populations is provided by Fisher *et al*. (2019). Here, both indirect phenotypic effects (the effects of the phenotypes of competitors on the focal individuals’ phenotype and fitness) and IGEs of territorial neighbours on the timing of reproduction in North American red squirrels (*Tamiasciurus hudsonicus*) were investigated. Squirrels create food caches (‘middens’) in the centre of their territories. Among females, superior competitors store more food during autumn and winter, and breed earlier relative to weak competitors. Importantly, more competitive individuals should gain at the expense of less‐competitive neighbours. There was some evidence to support this, with indirect phenotypic effects of neighbours contributing significantly to variation in the parturition date (date of giving birth) among focal females at high population densities (but not at low population densities). Despite available pedigree data, it could not be shown conclusively that these indirect phenotypic effects themselves had a genetic basis (as expected if competitive ability is heritable). This is because, while estimates of indirect genetic effects suggested moderately large effect sizes, they were accompanied by very high levels of statistical uncertainty. Although it may be very difficult to obtain precise estimates in any single wild population, further studies of territorial species employing similar methodology would be highly valuable. This would enable meta‐analyses to assess if and to what extent social competition among individuals within heterogeneous environments can constrain microevolution through indirect genetic effects.

## VI. CONCLUSIONS

1. In this review we have highlighted the importance of social competition‐induced phenotype–environment correlations in ecology and evolution. It is a fundamental tenet of evolutionary theory that phenotypes interact with the environment to generate variation in fitness – indeed this *is* natural selection (Darwin, 1859). When we study selection we typically conceptualise phenotype, fitness and (extrinsic) environment as distinct but correlated entities linked by causal processes. Phenotypes influence fitness (i.e. selection) while environments can influence fitness directly but also *via* effects on phenotype (plasticity). However, we commonly neglect the possibility that phenotype–environment correlations can also reflect causal effects of phenotype on environment (Edelaar & Bolnick, 2012, 2019; Saltz & Nuzhdin, 2014; Saltz, 2019). This likely represents an important omission. In particular, social competition may be underappreciated as a common cause of phenotype–environment correlations.
2. Social competition over preferred environments is widespread and variation in traits influencing competition outcomes will generate positive feedback loops that amplify fitness variation. As such, competition for preferred environments can act as an important driver of among‐individual fitness variation in natural populations. In this scenario the convenient but simplistic separation of individual quality and environmental quality becomes untenable.
3. How important this phenomenon is remains an open empirical question. Progress in answering it may require increased incorporation of experimental manipulation into ecological studies. This will be the most effective route to establishing the causation of observed phenotype–environment correlations.
4. We conclude that phenotype–environment correlations arising from competition within heterogeneous environments may constrain phenotypic evolution. Specifically, genetically determined differences in the ability of individuals to obtain preferred habitat under competition will generate indirect genetic effects on resource acquisition that, in turn, reduce the capacity of resource‐dependent life‐history traits to evolve under directional selection (Wilson, 2014; Fisher & McAdam, 2019). The latter phenomenon could explain why in many populations among‐individual fitness variation is maintained (e.g. Bonnet *et al*., 2017; Pujol *et al*., 2018). Quantitative genetic methods based on pedigrees or relatedness inference from molecular data offer a practical way forward. The possibility that the evolution of resource‐dependent life‐history traits may be constrained by social competition is both relevant for fundamental research, and for applied research aiming to understand the adaptive potential of populations threatened by processes such as climate change.
